# Identification of Key lncRNA–mRNA Pairs and Functional lncRNAs in Breast Cancer by Integrative Analysis of TCGA Data

**DOI:** 10.3389/fgene.2021.709514

**Published:** 2021-08-20

**Authors:** Zhe Li, Zheng Qian, Fei Chen, Shujun Jiang, Lingjia Meng, Jinzhong Chen

**Affiliations:** ^1^Department of Breast Surgery, Shuguang Hospital Affiliated to Shanghai University of Traditional Chinese Medicine, Shanghai, China; ^2^Department of General Surgery, Putuo Hospital Affiliated to Shanghai University of Traditional Chinese Medicine, Shanghai, China; ^3^State Key Laboratory of Genetic Engineering, School of Life Sciences, Fudan University, Shanghai, China

**Keywords:** LINC01235, proliferation, prognosis, breast cancer, invasion

## Abstract

Long non-coding RNAs (lncRNAs) play an important role in many diseases and are involved in the post-transcriptional regulatory network of tumors. The purpose of this study is to mine new lncRNA–mRNA regulatory pairs and analyze the new mechanism of lncRNA involvement in breast cancer progression. Using breast cancer miRNA and mRNA expression profiling from The Cancer Genome Atlas (TCGA), we identified 59 differentially expressed lncRNAs, 88 differentially expressed miRNAs, and 1,465 differentially expressed mRNAs between breast cancer tissue and adjacent normal breast cancer. Whereafter, four candidate lncRNAs (FGF14-AS2, LINC01235, AC055854.1, and AC124798.1) were identified by the Kaplan–Meier (K–M) plotter. Furthermore, we screened the hub lncRNA (LINC01235) through univariate Cox analysis, multivariate Cox analysis, and qPCR validation, which was significantly correlated with breast cancer stage, ER status, and pathological N. Subsequently, 107 LINC01235-related mRNAs were obtained by combining differentially expressed miRNAs, differentially expressed mRNAs, and LINC01235 targeting miRNAs and mRNAs. The protein–protein interaction (PPI) network was established by Cytoscape software, and 53 key genes were screened. Function and pathway enrichment showed that LINC01235-related key genes might be involved in the process of cell differentiation, cell proliferation, and p53 signal pathway. In addition, LINC01235 has been confirmed to regulate the proliferation, migration, and invasion of MCF-7 cells in *in vitro* experiments. Furthermore, we screened three mRNAs (ESR1, ADRA2A, and DTL) associated with breast cancer drug resistance from key genes. Through RNA interference experiments *in vitro* and correlation analysis, we found that there was a negative feedback mechanism between LINC01235 and ESR1/ADRA2A. In conclusion, our results suggest that LINC01235-ESR1 and LINC01235-ADRA2A could serve as important co-expression pairs in the progression of breast cancer, and LINC01235 plays a key role as an independent prognostic factor in patients with breast cancer. The findings of this work greatly increase our understanding of the molecular regulatory mechanisms of lncRNA in breast cancer.

## Introduction

Long non-coding RNAs (lncRNAs) are a class of RNA molecules that are over 200 nucleotides in length, which have no protein-coding function but have epigenetic regulation and other biological functions (Peng et al., 2017). Currently, mounting evidences have demonstrated that lncRNAs and mRNAs played critical roles in various physiological processes, such as cell activity, gene regulation, and so on (Fukushima and Kida, 2019; De Troyer et al., 2020; Labonté et al., 2020). Therefore, dissecting how lncRNAs regulate tumor progress attracts great interest from researchers (Majidinia and Yousefi, 2020). In breast cancer (BRCA), high levels of BCRT1, SPRY4-IT1, RP11-19E11, and FAM83H-AS1 were associated with clinical stage and prognosis (Giro-Perafita et al., 2020; Han et al., 2020; Liang et al., 2020; Song et al., 2020). These studies indicated that lncRNAs could be regarded as valuable biomarkers and therapeutic targets for cancer therapy.

Breast cancer is one of the common malignant tumors among women and ranks first among malignant tumors in women (DeSantis et al., 2019). In China, the age of patients suffering from breast cancer is getting lower (Rossi et al., 2019). However, treatments may temporarily control tumor growth but very often lead to a chemotherapy resistance status (Tang et al., 2016). So, it is very important and meaningful to find new markers or therapeutic targets. LINC01235 is located on the chromosome 9 p23 and expressed in a variety of cancer tissues. It has been reported that the expression of LINC01235 was specific in a specific environment or tissue and had a characteristic expression pattern in tumors (Tang et al., 2019; Xu et al., 2019). In gastric cancer, LINC01235 can promote gastric cancer cell metastasis through EMT and be used as a biomarker of prognosis (Tan et al., 2020). In breast cancer, the expression of LINC01235 was analyzed (Vishnubalaji et al., 2019). The high expression of LINC01235 was associated with poor prognosis of breast cancer patients (Li et al., 2021; Zhang K. et al., 2021). These results are consistent with the data we analyzed. However, the regulation of LINC01235 on the progression of breast cancer has not been reported. In addition, it is strange that the low expression of LINC01235 in breast cancer has a good prognosis, while the high expression of LINC01235 has a poor prognosis. At present, there is no relevant experimental data to explain this problem.

In this study, we explored the differentially expressed lncRNAs in the breast cancer based on The Cancer Genome Atlas (TCGA) dataset. Combined with the Kaplan–Meier (K–M) plotter analysis, protein–protein interaction (PPI) network, expression analysis, and RNA interference experiment, we found the most relevant mRNA (ESR1 and ADRA2A) of LINC01235, and there was an obvious negative feedback between LINC01235 and two mRNAs. Moreover, a series of interference analyses and validation experiments in MCF-7 cells were used to analyze the effect of LINC01235 on the progression of breast cancer. All results may be used to provide data reference for the study of LINC01235 to regulate breast cancer deterioration.

## Materials and Methods

### Patients’ Enrollment From TCGA–BRCA

Our research excluded any samples that had missing or insufficient data on age, TNM stage, OS time, lymph node metastases, and ER status. We retained both RNA-Seq and clinical data, which we used for further investigation. The detailed clinical and pathological characteristics of the study population are shown in Table 1. A total of 1,028 patients were pathologically diagnosed as having breast cancer. The median age of all patients was 52 years (26–91 years). Majority of the patients were white (68.58%) with high histological grade (91.21%).

**TABLE 1:** Clinical pathological characteristics of 1,028 patients with breast cancer.

Parameter	Subtype	Patientsn (%)
Age (Years)	26 to ≤52	344 (33.46%)
	52 to ≤91	684 (66.54%)
Race	White	705 (68.58%)
	Black or African American	173 (16.83%)
	Asian	56 (5.45%)
	Not reported	94 (9.14%)
Pathologic stage	Stage i	170 (16.54%)
	Stage ii	584 (56.82%)
	Stage iii	231 (22.47%)
	Stage iv	19 (1.85%)
	Missing	24 (2.33%)
Pathologic T	T1	267 (25.97%)
	T2	597 (58.08%)
	T3	125 (12.16%)
	T4	36 (3.5%)
	TX	3 (0.29%)
Pathologic M	M0	855 (83.17%)
	M1	21 (2.04%)
	MX	152 (14.79%)
Pathologic N	N0	474 (46.11%)
	N1	346 (33.65%)
	N2	117 (11.38%)
	N3	71 (6.91%)
	NX	20 (1.95%)
Vital Status	Alive	885 (86.09%)
	Dead	143 (13.91%)

### Screening of Differentially Expressed RNAs

Breast cancer RNA-seq sequencing expression profile data, miRNA sequencing (miRNA-seq) expression profile data, and clinical data were downloaded from the open access data of the TCGA database.^1^ We obtained 30 adjacent normal and 300 tumor sample dates for mRNA and lncRNA analysis and obtained 10 adjacent normal and 357 tumor sample dates for miRNA analysis. The clinical information of the cases was also collected, including age, stage, pathologic tumor (pathologic T), pathologic node (pathologic N), pathologic metastasis (pathologic M), lymph node metastases, and ER status. Differentially expressed mRNAs, differentially expressed miRNAs, and differentially expressed lncRNAs between the breast cancer tumor tissues and the normal samples were screened using the limma package in R, with the criterion of log2| Fold Change| > 1.5, adjusted *p*-value < 0.01, and FDR (false discovery rate) < 0.05.

### Kaplan–Meier Survival Curve Analysis

To investigate the impact of the expression levels of differentially expressed RNAs on the prognostic survival of breast cancer patients, K--M survival curve analysis was performed using the online tool ‘‘GEPIA.’’^2^ The statistical significance was set at *p* < 0.05. Then, the data obtained from the analysis are verified by Starbase^3^.

### RNA Extraction and Real-Time Quantitative Reverse Transcription Polymerase Chain Reaction

A total of 35 breast cancer tissues and 35 adjacent normal tissues were collected. This study was approved by the Ethical Committee of Hospital and was conducted in accordance with the Declaration of Helsinki. In addition, each patient offered written informed consent. All tissues were immediately stored at −80°C.

Total RNA of tissue was extracted using the RNeasy Mini Kit (Qiagen, Germany) according to the manufacturer’s protocol. Then, the extracted RNA was qualitatively controlled and quantified by Nanodrop. The equivalent amount of RNA was reverse transcribed into cDNA with Golden star TM RT6 cDNA synthesis KIT ver.2 (TsingKe, Beijing, China). LncRNA and mRNA expression was then examined by real-time quantitative reverse transcription polymerase chain reaction (qRT-PCR) with Master qPCR Mix (SYBR GREEN 1) (TsingKe, Beijing, China) and Biosystems 7,500 Fast Real-time PCR System. GAPDH expression was used as endogenous control. The primers were designed using Primer 5.0 software (Table 2). The 2^–ΔΔ*Ct*^ method was used to calculate the levels of lncRNA expression and mRNA expression. Excel was used to analyze the qRT-PCR data, and each reaction was performed in triplicate. Two groups were performed using *t*-test (*p* < 0.05).

**TABLE 2:** The sequences of primers.

Gene symbol	Primer	Sequences (5′-3′)
LINC01235	Forward primer	GCCTACCTTACCTGTGGCTC
	Reverse primer	CGTTGACCTGTGAGAGACCC
FGF14-AS2	Forward primer	AGTTCCAGTTACCATCTTCA
	Reverse primer	AGGTTCATAGTTGCCAGAC
AC055854.1	Forward primer	CACTGTCTCAGGTCACGCTT
	Reverse primer	GTAGGGTGAGGGGAGGGAAT
AC124798.1	Forward primer	CACGTGGGCCAGAAAAAGTG
	Reverse primer	GAGGGAAGGTGGAACTCAGC
ESR1	Forward primer	TGAACCATCACTGAGGTGG
	Reverse primer	TGGATCTGATGCAGTAGGG
ADRA2A	Forward primer	AGAAGTGGTACGTCATCTCG
	Reverse primer	ATCATGATGAGGCAGGGAG

### Prediction of lncRNA Subcellular Localization

Long non-coding RNA sequences were obtained from LNCipedia.^4^ lncRNA cellular localization was predicted by its sequence using lncLocator.^5^ Localization is determined by the scores of different subcellular locations.

### Protein–Protein Interaction Network Construction

Three databases (Starbase,^6^ DIANA tools,^7^ and miRTarbas^8^) were used to verify the miRNA--protein coding gene mRNA and miRNA--lncRNA regulation correlation. Only the overlapping target genes were identified to enhance the reliability of the bioinformatics analysis. Then, overlapping target genes were used to construct the PPI network. The PPIs were analyzed using the STRING database (STRING^9^) and a combined score >0.5 was used as the cutoff criterion. Additionally, Cytoscape was used to construct and visualize the PPI network.

### Cox Proportional Regression Model Based on Differentially Expressed lncRNAs

In order to analyze the independent effect of individual lncRNA on the overall survival (OS) of breast cancer patients, we used online tools (SangerBox tools^10^) to carry out univariate and multivariate Cox proportional regression analysis. We constructed a Cox proportional hazard regression model to calculate the value at risk of each patient. Risk score = expression of lncRNA1 × β_*lncRNA*__1_ + expression of lncRNA2 × β_*lncRNA*__2_ + …… + expression of lncRNAn × β_*lncRNAn*_. β represents the multivariate Cox regression coefficient. Then, we calculated the survival rate of the high-risk group and the low-risk group, and drew 1-, 3-, and 5-year ROC (survival receiver operating characteristic) curves to test the feasibility of the predictive ability of the model.

### Gene Ontology and Kyoto Encyclopedia of Genes and Genomes Analysis

Functional enrichment analysis of lncRNA-target genes was performed using Metascape. All statistically enriched terms [Gene Ontology (GO) and Kyoto Encyclopedia of Genes and Genomes (KEGG)] were identified based on accumulative hypergeometric *p* values.

### Cell Culture and Cell Transfection

MCF-7 was purchased from the Beijing Beina Chuanglian Biotechnology Research Institute (Beijing, China). MCF-7 was cultured in Roswell Park Memorial Institute 1,640 medium (Gibco) supplemented with 10% fetal bovine serum (Thermo Scientific), 100 U/ml penicillin, and 100 μg/ml streptomycin under a humidified atmosphere of 5% CO_2_ at 37°C. The cell lines have been identified and the fourth passage cells of MCF-7 were used to perform experiments in this study. MCF-7 cells were transfected with 50 nM siRNA (si-LINC01235, si-ESR1, and siADRA2A; Ribobio, Smart Silencer), pCDH-LINC01235 (Ribobio, Smart Silencer), and their NC controls for 48 h using Lipofectamine 3,000 reagent (Invitrogen, Shanghai, China) according to the manufacturer’s instructions.

### Cell Counting Kit-8 Assays

Cell counting Kit-8 (CCK8) (Sigma, United States) was used to detect the proliferation of MCF-7 cells in 96-well plates. A total of 3,000 cells/well were incubated for 24 and 48 h. All cells were incubated with CCK-8 reagent (10 μl per well) for 3 h, and the absorbance of each well was detected by enzyme labeling instrument made by Thermo Company at 450 nm wavelength. Each experiment was conducted three times.

### Wound-Healing Assay

MCF-7 cells were inoculated in a petri dish (96 wells, Corning, United States) with 5 × 10^5^ cells per well. The temperature of the culture medium was 37°C and the concentration of CO_2_ was 5%. Then, the fusion cell monolayer was scratched with the tip of a 200-ml aseptic pipette, and Opti-MEM reduced serum medium (Gibco, United States) was added. The scratches were recorded by a microscope at 0, 24, and 48 h later. The closure rate was evaluated by ImageJ software.

### Transwell Assay

After 48 h of transfection, MCF-7 cells were collected to prepare single-cell suspension. The MCF-7 cell suspension (3 × 10^3^ cell/well) was added to the upper chamber (Corning, United States), and the Roswell Park Memorial Institute 1,640 medium (RPMI-1640, 20% fetal bovine serum) was added to the 24-well plate in the lower chamber. The upper chamber was coated with matrix glue. After 24 h, the cells were fixed with 4% paraformaldehyde (Sigma, United States) and stained with 1% crystal violet (Sigma, United States). Cells were observed and counted under a light microscope (Olympus, Japan).

### Statistical Analysis

SPSS and GraphPad Prism were used to analyze the experimental data. Student’s *t*-test or ANOVA variance test was used for comparison. All variables were expressed as mean ± standard deviation (SD), which were obtained from three independent experiments. *p* < 0.05 was a significant difference.

## Results

### Differentially Expressed lncRNAs, miRNAs, and mRNAs in Breast Cancer Patients

**FIGURE 1:**
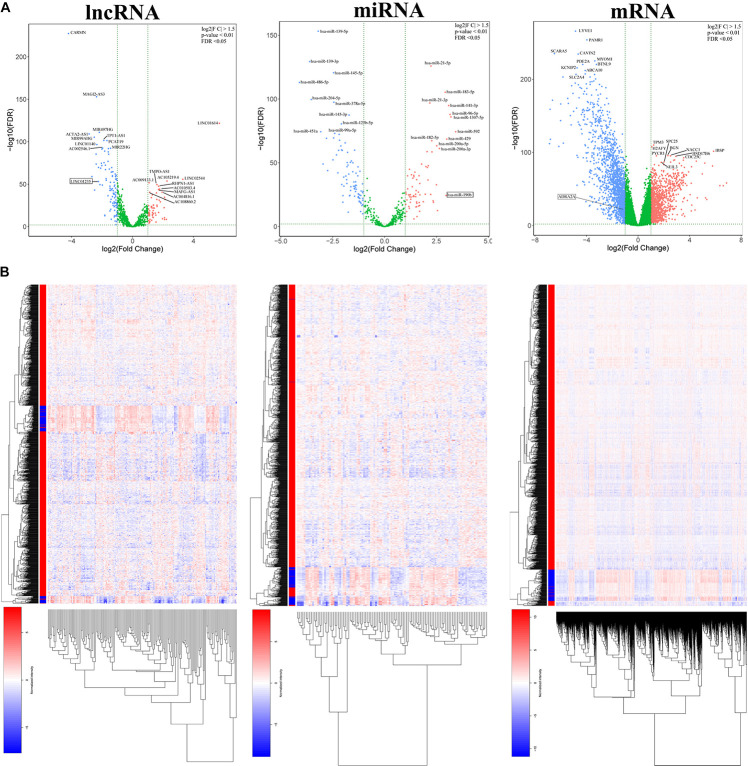
**(A)** Volcano plots of differentially expressed RNAs (lncRNA, miRNA, and mRNA) in the The Cancer Genome Atlas (TCGA) database of breast cancer. The red dots represent upregulated RNAs, and the blue dots represent downregulated RNAs. The green dots represent genes with no significant difference. **(B)** Heatmap of differentially expressed RNAs (lncRNA, miRNA, and mRNA) in the TCGA database of breast cancer.

In order to study the potential role of RNAs (lncRNAs, miRNAs, and mRNAs) in the development of breast cancer, we identified the differentially expressed RNAs using the limma package in R. The RNA expression profile data of breast cancer patients were downloaded from the open access data of the TCGA database. By setting the criteria thresholds to log2| Fold Change| > 1.5, adjusted *p*-value < 0.01, and FDR < 0.05, a total of 59 differentially expressed lncRNAs (24 upregulated and 35 downregulated) (Table 3), 88 differentially expressed miRNAs (42 upregulated and 46 downregulated) (Table 4), and 1,464 differentially expressed mRNAs (520 upregulated and 944 downregulated) (Table 5) were identified. Volcano plots (Figure 1A) and heatmap (Figure 1B) showed the number of differentially expressed RNAs identified from the TCGA database.

**TABLE 3:** Top10 (up- and down-regulated) of differentially expressed lncRNAs in normal tissues and breast cancer tissues.

ID	Symbol	logFC	AveExpr	*t*	*p*-Value
**Up regulation**					
ENSG00000204049	AL391421.1	1.960	−0.039	6.401	2.21E-10
ENSG00000227036	LINC00511	2.068	1.255	10.253	1.03E-23
ENSG00000218416	PP14571	2.094	1.086	6.844	1.22E-11
ENSG00000253125	AC055854.1	2.095	0.459	6.058	1.84E-09
ENSG00000265415	AC099850.3	2.098	0.270	12.976	3.80E-36
ENSG00000267751	AC009005.1	2.127	0.225	12.458	1.31E-33
ENSG00000228630	HOTAIR	2.216	0.872	6.119	1.27E-09
ENSG00000254973	AC105219.4	2.257	0.163	16.541	1.42E-55
ENSG00000261039	LINC02544	3.297	0.393	16.893	1.18E-57
ENSG00000230838	LINC01614	5.693	1.161	26.773	1.96E-124
**Down regulation**					
ENSG00000249669	CARMN	−4.206	0.747	−41.050	1.31E-231
ENSG00000180139	ACTA2-AS1	−2.854	0.985	−25.018	1.03E-111
ENSG00000196167	COLCA1	−2.671	0.273	−17.435	6.43E-61
ENSG00000215386	MIR99AHG	−2.517	1.231	−24.477	7.65E-108
ENSG00000234899	SOX9-AS1	−2.491	0.948	−14.640	8.26E-45
ENSG00000272143	FGF14-AS2	−2.394	−0.071	−21.585	1.07E-87
ENSG00000229847	EMX2OS	−2.388	0.598	−19.807	7.22E-76
ENSG00000234456	MAGI2-AS3	−2.365	3.204	−31.014	5.89E-156
ENSG00000267272	LINC01140	−2.346	0.470	−23.226	5.17E-99
ENSG00000270547	LINC01235	−2.242	1.199	−16.445	5.20E-55

**TABLE 4:** Top10 (up- and down-regulated) of differentially expressed miRNAs in normal tissues and breast cancer tissues.

Symbol	logFC	AveExpr	*t*	PValue	FDR
**Up regulation**					
hsa-miR-200a-5p	2.5124	8.8659	18.1566	2.73E-65	4.85E-64
hsa-miR-184	2.5491	2.7064	7.4899	1.33E-13	3.15E-13
hsa-miR-200a-3p	2.6410	7.7297	17.8111	3.70E-63	6.10E-62
hsa-miR-183-5p	2.9236	13.5554	24.5103	6.47E-108	4.80E-106
hsa-miR-190b	2.9605	3.0982	10.7373	9.58E-26	3.77E-25
hsa-miR-429	2.9959	6.3372	19.0099	1.18E-70	2.50E-69
hsa-miR-141-3p	3.0892	9.6728	22.9897	3.17E-97	1.41E-95
hsa-miR-96-5p	3.1552	4.7125	21.9740	3.13E-90	1.27E-88
hsa-miR-1307-5p	3.1966	7.3873	21.6791	3.17E-88	1.09E-86
hsa-miR-592	3.4271	1.2954	19.9167	1.70E-76	4.71E-75
**Down regulation**					
hsa-miR-486-5p	−4.0994	5.2776	−25.5942	1.13E-115	1.01E-113
hsa-miR-139-3p	−3.6041	2.6593	−27.8961	1.89E-132	4.20E-130
hsa-miR-204-5p	−3.5308	0.9813	−23.6935	3.83E-102	2.44E-100
hsa-miR-139-5p	−3.1947	5.2511	−31.1418	1.39E-156	6.17E-154
hsa-miR-451a	−3.0756	7.0965	−19.9251	1.49E-76	4.43E-75
hsa-miR-5683	−2.9633	2.3067	−15.7227	8.50E-51	9.23E-50
hsa-miR-144-5p	−2.7977	5.1749	−19.1086	2.77E-71	6.17E-70
hsa-miR-1247-3p	−2.7420	3.8113	−14.7795	1.53E-45	1.36E-44
hsa-miR-452-5p	−2.4861	4.7375	−16.3033	3.88E-54	4.80E-53
hsa-miR-145-5p	−2.4557	10.3201	−26.6543	2.35E-123	2.62E-121

**TABLE 5:** Top10 (up- and down-regulated) of differentially expressed mRNAs in normal tissues and breast cancer tissues.

Symbol	logFC	AveExpr	t	*p*-Value	FDR
**Up regulation**					
COMP	4.5911	5.6252	13.6328	1.77E-39	1.38E-38
SLC24A2	4.9779	1.0255	19.3263	9.37E-73	2.27E-71
PLPP4	5.1782	1.7559	21.5819	1.12E-87	3.83E-86
MMP1	5.2342	1.9926	13.6401	1.62E-39	1.27E-38
COL11A1	5.3334	5.9023	13.1931	3.10E-37	2.24E-36
MMP11	5.7762	6.9196	20.9930	1.05E-83	3.30E-82
IBSP	5.8592	−0.4205	23.9461	4.48E-104	2.34E-102
MMP13	6.3473	2.6126	16.6949	1.76E-56	2.47E-55
CST1	6.6093	0.7129	16.6713	2.43E-56	3.37E-55
COL10A1	6.8648	5.663608	17.3076	3.81E-60	6.08E-59
**Down regulation**					
ADH1B	−6.8751	2.8739	−25.4161	1.41E-114	9.33E-113
TUSC5	−6.8274	0.5241	−31.8335	3.98E-162	9.37E-160
ADIPOQ	−6.8086	2.1216	−26.2480	1.32E-120	1.00E-118
CIDEC	−6.6436	0.7323	−33.4936	1.16E-174	3.95E-172
SCARA5	−6.4982	0.2681	−42.1088	1.71E-239	8.72E-236
FABP4	−6.1494	3.8951	−24.8773	1.05E-110	6.41E-109
PLIN1	−6.0173	3.2862	−29.7342	2.32E-146	3.18E-144
GPD1	−6.0055	2.6358	−32.8030	1.93E-169	5.56E-167
AQP7	−5.8304	0.5324	−37.7592	6.58E-207	6.70E-204
PLIN4	−5.8014	4.3026	−25.5145	2.75E-115	1.85E-113

### Long Non-coding RNAs Associated With Prognosis and Clinical Parameters in Breast Cancer Patients

**FIGURE 2:**
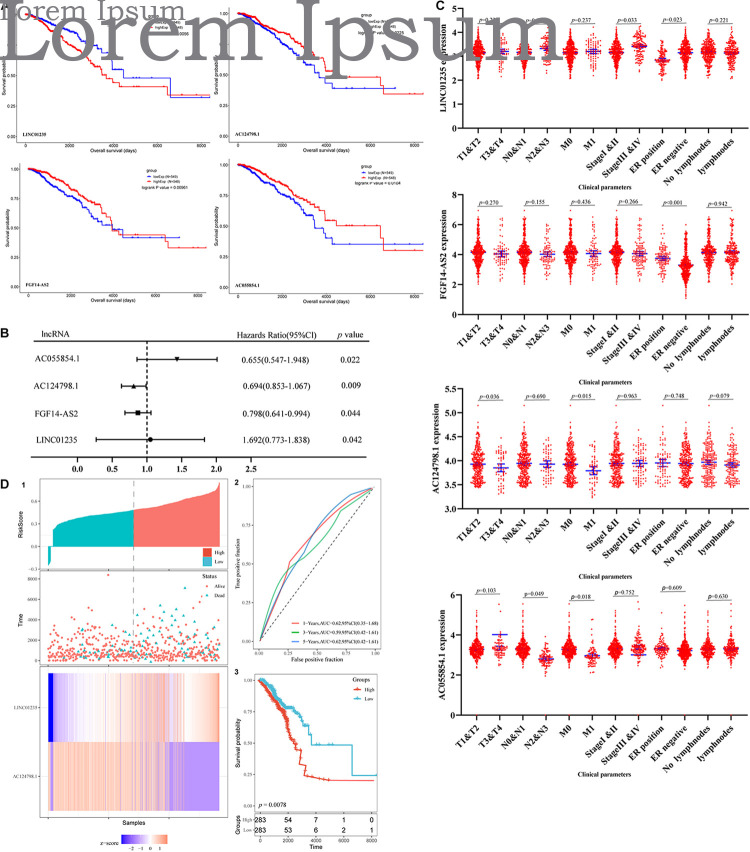
**(A)** Kaplan–Meier (K–M) analysis results of LINC01235, AC055854.1, FGF14-AS2, and AC124798.1, the four lncRNAs found to be associated with the prognosis of breast cancer. **(B)** Univariate Cox proportional hazards regression analysis of LINC01235, AC055854.1, FGF14-AS2, and AC124798.1 in breast cancer. **(C)** Correlation analysis of the expression of LINC01235, AC055854.1, FGF14-AS2, and AC124798.1 with clinicopathological parameters. **(D)** Prognostic risk score model analysis of LINC01235 and AC124798.1 in breast cancer patients. (1) Risk score distribution, patient survival status distribution, and heatmap of P LINC01235 and AC124798.1 expression by risk score. (2) The ROC curves for predicting survival in breast cancer patients by the risk score. (3) Kaplan–Meier curves for high-risk and low-risk groups.

The prognosis of breast cancer according to the expression of differentially expressed lncRNAs was performed based on the dates obtained from the TCGA database by GEPIA database. A total of 1,097 cases were included in analysis. Among the 59 deregulated lncRNAs, we found that the FGF14-AS2, LINC01235, AC055854.1, and AC124798.1 were statistically related to the OS in breast cancer patients (*p* < 0.05; Figure 2A). The high expression levels of LINC01235 predicted poor prognosis, while the other three lncRNAs FGF14-AS2, AC055854.1, and AC124798.1 were positively correlated with patient survival. These results suggested that the four lncRNAs might play important roles in the development of breast cancer. Meanwhile, the expression levels of four differentially expressed lncRNAs (FGF14-AS2, LINC01235, AC055854.1, and AC124798.1) were analyzed using a univariate Cox proportional hazards regression model. The analysis results are consistent with those of the K–M curve. FGF14-AS2, LINC01235, AC055854.1, and AC124798.1 were identified as prognostic factors of breast cancer patients (*p* < 0.05; Figure 2B).

**FIGURE 3:**
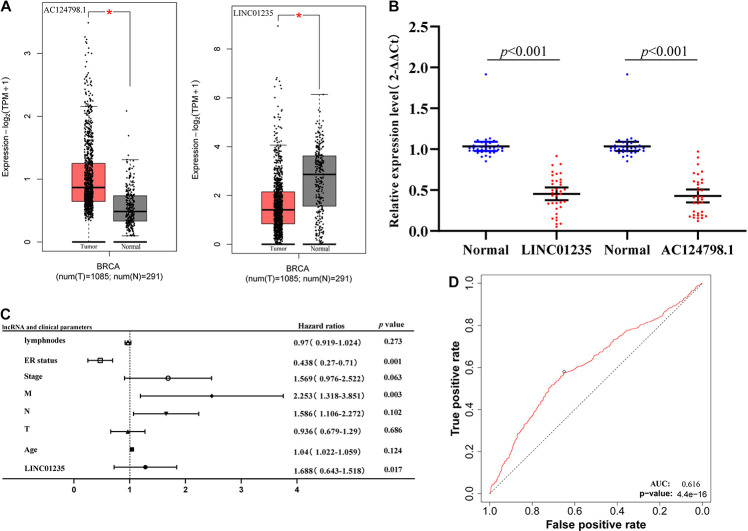
**(A)** The box plot shows the expression trend of LINC01235 and AC124798.1 in TCGA breast cancer using GEPIA (http://gepia2.cancer-pku.cn/#index). *p < 0.05. **(B)** Real-Time Quantitative Reverse Transcription Polymerase Chain Reaction (RT-qPCR) analysis was conducted to assess the expression levels of LINC01235 and AC124798.1 in 35 paired breast cancer tissues and their corresponding adjacent normal tissues. The data were shown as mean ± standard deviations. **(C)** Multivariate Cox analysis of LINC01235 expression and pathological parameters. **(D)** Receiver operating characteristic (ROC) curves of LINC01235.

Moreover, we evaluated the relationship among four lncRNA (FGF14-AS2, LINC01235, AC055854.1, and AC124798.1) expression levels and various clinicopathological parameters of breast cancer patients. Expression data and clinical characteristics were obtained from the TCGA–BRCA database. The results showed that the expression of FGF14-AS2, LINC01235, AC055854.1, and AC124798.1 was significantly correlated with tumor stage, pathologic TNM, and ER status (*p* < 0.05; Figure 2C). From the multivariate Cox regression analysis, LINC01235 and AC124798.1 were further screened from the above four lncRNAs with prognostic significance (Table 6). The hazard ratios of AC124798.1 (HR = 0.695*;* 95% CI, 0.848–1.064; *p* = 0.037) were less than 1; however, the hazard ratios of LINC01235 (HR = 1.688, 95% CI, 0.788–1.884; *p* = 0.017) were greater than 1. Furthermore, LINC01235 and AC124798.1 were used to construct a risk score model. The risk scores for samples from TCGA were calculated using the formula: risk score = 0.2036 × expression of LINC01235–0.0551 × expression of AC124798.1. On the basis of the median risk score, breast cancer patients were divided into a high-risk and a low-risk group. The risk curve, scatterplot, and K–M analysis showed that the high-risk group displayed worse OS (Figure 2D-1,2). In order to confirm our findings, we calculated the risk score for the testing sets including GSE21653 (*n* = 252) and GSE42568 (*n* = 104). With the same cutoff, the patients from each testing set were separately classified into low-risk and high-risk groups. Similar to the findings obtained from the TCGA–BRCA set, patients in the high-risk group had shorter survival time than patients in the low-risk group (Supplementary Figure 1A). Besides, ROC curve analysis showed that the AUC of 1, 3, and 5 years was 0.62, 0.59, and 0.62, respectively (Figure 2D-3). All the results indicated that the risk score model has prognostic value. Therefore, these above results confirmed that LINC01235 and AC124798.1 have significant prognostic significance in breast cancer patients.

**TABLE 6:** Multivariate cox regression analysis of FGF14-AS2, LINC01235, AC055854.1, and AC124798.1 associated with survival in breast cancer patients.

	Coefficient	HR	SE	*P*-value
AC055854.1	0.055	1.056	0.328	0.867
AC124798.1	−0.055	0.695	0.058	0.037
FGF14-AS2	−0.221	0.802	0.112	0.148
LINC01235	0.204	1.688	0.222	0.017

### Expression Validation of LINC01235 and AC124798.1 in Breast Cancer Tissues

As shown in Figure 3A, the bioinformatics analysis results showed that the expression level of LINC01235 was downregulated, and AC124798.1 was significantly increased compared to the normal tissues. To analyze the accuracy and reliability of present bioinformatics analysis, qPCR was used to evaluate LINC01235 and AC124798.1 expression level in 35 breast cancer tissues and 35 adjacent tissues. We found that only LINC01235 was consistent with bioinformatics analysis results. The expression of LINC01235 in breast cancer tissues was significantly downregulated when compared to breast normal tissues (*p* < 0.001; Figure 3B). Specifically, it was strange that LINC01235 is downregulated in breast cancer tissues when compared to normal specimens; however, its low expression correlates with a favorable prognosis. This may be the reverse regulation of LINC01235 in the regulation of breast cancer, which negatively affects the progression of breast cancer by regulating another factor. The specific mechanism remains to be further studied.

As shown in Figure 2C we have analyzed the relationship between the expression level of LINC01235 and various clinical parameters of breast cancer patients. The analysis results showed that increased expression of LINC01235 was significantly correlated with pathological N (*p* < 0.05), tumor stage (*p* < 0.05), and ER status (*p* < 0.05). In addition, univariate analysis revealed that LINC01235 expression, pathologic TNM (*p* < 0.05), tumor stage (*p* < 0.05), and ER status (*p* < 0.05) were significantly correlated with the OS of breast cancer patients (Table 7). Multivariate analysis showed that LINC01235 expression might be an independent factor for the prognosis of breast cancer patients (Figure 3C and Table 8). Meanwhile, the ROC curve AUC of LINC01235 expression for predicting survival was 0.616 (Figure 3D), which indicated that LINC01235 possessed the potential prognostic ability of breast cancer. In order to further verify the clinical value of LINC01235, we combined the two datasets GSE21653 and GSE42568 as independent testing sets for ROC. The area under receiver operating characteristic (AUROC) was 0.8124 (*p* < 0.0001; Supplementary Figure 1B). These results indicated that breast cancer patients with high levels of LINC01235 expression are more likely to promote breast cancer progression.

**TABLE 7:** Univariate analysis of LINC01235 in breast cancer patients.

Clinical characteristic	HR	LOWER	UPER	P
Age	1.033	1.015	1.051	0.079
T	1.418	1.086	1.852	0.010
N	1.712	1.361	2.155	<0.001
M	2.675	1.609	4.449	<0.001
Stage	1.941	1.421	2.651	<0.001
ERstatus	0.556	0.346	0.896	0.016
lymph nodes	1.047	1.012	1.083	0.308
LINC01235	1.792	0.773	1.838	0.042

**TABLE 8:** Multivariate survival analysis of LINC01235 in breast cancer patients.

Clinical characteristic	HR	LOWER	UPER	P
LINC01235	1.688	0.643	1.518	0.017
Age	1.04	1.022	1.059	0.124
T	0.936	0.679	1.29	0.686
N	1.586	1.106	2.272	0.102
M	2.253	1.318	3.851	0.003
Stage	1.569	0.976	2.522	0.063
ER status	0.438	0.27	0.71	0.001
lymph nodes	0.97	0.919	1.024	0.273

### Construction of LINC01235-Related mRNA PPI Network

**FIGURE 4:**
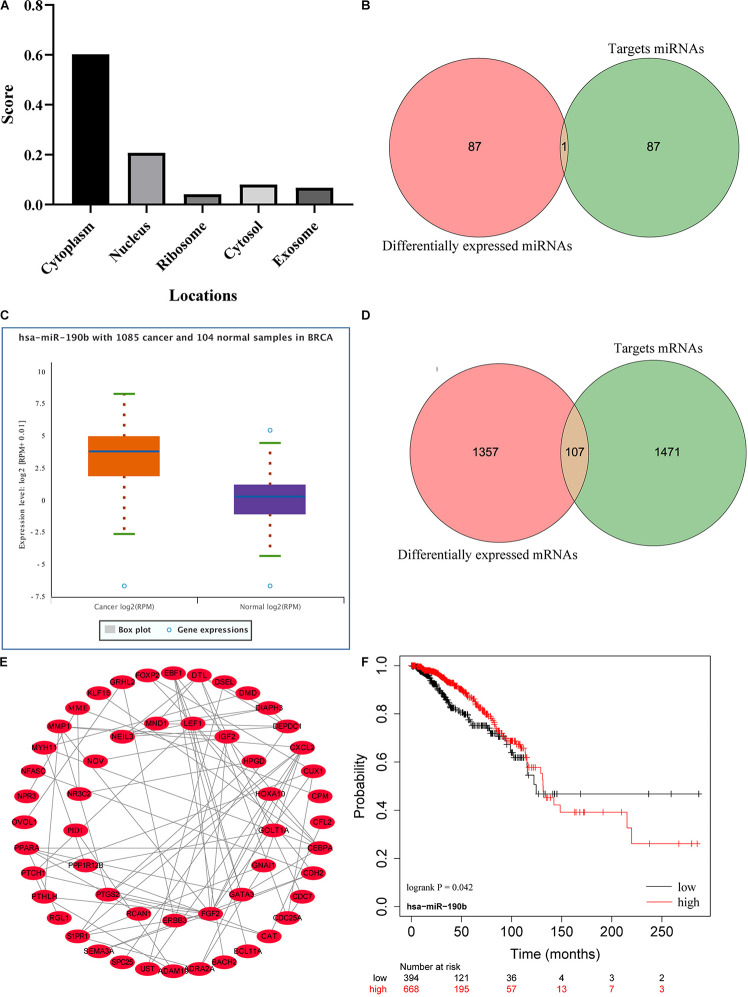
**(A)** Subcellular localizations of LINC01235 and FGF14-AS2 by using lncLocator (http://www.csbio.sjtu.edu.cn/bioinf/lncLocator/). **(B)** Venn diagram shows the overlapping miRNAs of LINC01235 targeting miRNAs and differentially expressed miRNAs. **(C)** The box plot shows the expression trend of miR-190b in TCGA breast cancer using Starbase (http://starbase.sysu.edu.cn/). **(D)** Venn diagram shows the overlapping mRNAs of miR-190b targeting mRNAs and differentially expressed mRNAs. **(E)** Protein–protein interaction (PPI) network of the hub genes. The regulatory relationship of hub genes was visualized using Cytoscape. **(F)** Kaplan–Meier analysis results of miR-190b in breast cancer.

The cellular localization of lncRNAs determines their molecular mechanism. We first explored the subcellular localization of those lncRNAs by using lncLocator. LINC01235 was identified to be located in the cytoplasm (Figure 4A; score = 0.60), which indicated that LINC01235 was more likely to exert their biological functions through ceRNA mechanism. The target miRNAs of LINC01235 were analyzed by DIANA tools (Supplementary Table 1). Then, we summarized the same miRNAs between target miRNAs of LINC01235 and differentially expressed miRNAs from TCGA. Only miR-190b was the overlap miRNA (Figure 4B), which was significantly expressed compared with non-tumor tissues in TCGA breast cancer (Figure 4C). Whereafter, the target mRNAs of miR-190b were also analyzed by DIANA tools (Supplementary Table 2). Following the same method, we obtained 107 overlap mRNAs between targeted mRNAs and differentially expressed mRNAs (Figure 4D and Supplementary Table 3). Then, 53 hub mRNAs were obtained by constructing the PPI network of overlap mRNAs (Figure 4E and Supplementary Table 4). Besides, the prognosis of breast cancer according to the expression of miR-190b was determined. The results showed that miR-190b was statistically related to the OS in breast cancer patients (*p* < 0.05; Figure 4F).

### Functional Enrichment Analysis of LINC01235-Related Hub Target Genes

**FIGURE 5:**
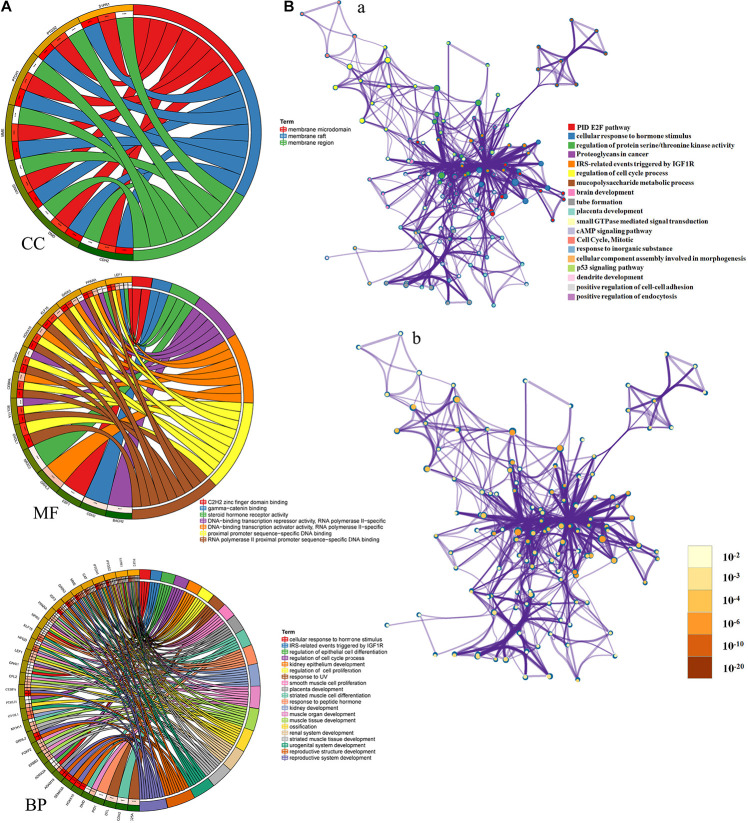
**(A)** Gene Ontology (GO) enrichment analysis of hub mRNAs in the PPI network was performed using Sangerbox (http://sangerbox.com/). CC, Cell components; MF, Molecular function; BP, Biological processes. **(B)** Interaction network of GO and Kyoto Encyclopedia of Genes and Genomes (KEGG) enriched terms using Metascape. **(a)** Colored by cluster ID, where nodes that share the same cluster ID are typically close to each other; **(b)** colored by *p*-value, where terms containing more genes tend to have a more significant *p*-value.

To better understand these LINC01235-related hub target mRNAs, we first performed GO functional annotation and KEGG pathway enrichment analysis by Enricher database and Metascape. The results showed that LINC01235 was also obviously associated with cellular response to hormone stimulus, regulation of protein serine/threonine kinase activity, IRS-related events triggered by IGF1R, regulation of epithelial cell differentiation, regulation of cell cycle process, regulation of epithelial cell proliferation, and regulation of epithelial cell proliferation. For KEGG pathway enrichment analysis, these LINC01235-related hub target mRNAs were significantly enriched in cAMP signaling pathway, PID E2F pathway, p53 signaling pathway, and proteoglycans in cancer (Figure 5A, CC, MF, and BP). To further capture the relationships between the terms, a subset of enriched terms has been selected and rendered as a network plot. Each node in the network represents an enriched term and is colored first by its cluster ID (Figure 5B-a) and then by its *p*-value (Figure 5B-b). By the color and size of each point, we can clearly identify biological functions or pathways that are significantly enriched, as well as associations with other biological functions or pathways.

### LINC01235 Contributes to MCF-7 Cell Proliferation, Migration, and Invasion

**FIGURE 6:**
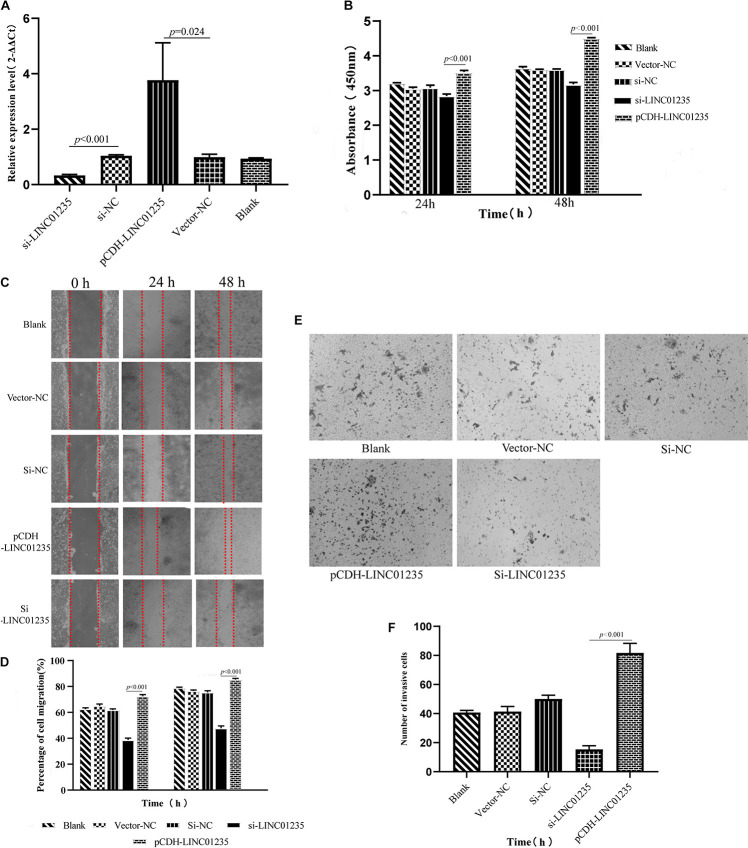
**(A)** LINC01235 knockout and overexpressed efficiency in MCF-7 cells analyzed by qPCR. **(B)** The effects of LINC01235 knockout and overexpressed on the proliferation activity of MCF-7 cells measured using the CCK-8. **(C,D)** The effects of LINC01235 knockout and overexpressed on MCF-7 cell migration measured using wound-healing assay. **(E,F)** The effects of LINC01235 knockout and overexpressed on MCF-7 cell invasion using transwell assay.

In order to further analyze the biological function of LINC01235, we used siRNA and overexpression plasmids to alter the expression level of LINC01235 in MCF-7 cells and analyzed the effect of LINC01235 on MCF-7 cell proliferation, migration, and invasion. qPCR results revealed successful transfection of the overexpression plasmid (pCDH-LINC01235) and si-LINC01235 (Figure 6A). The effect of LINC01235 on cell proliferation was detected by the CCK-8 assay. Compared with the negative control (NC) group, pCDH-LINC01235 significantly promoted MCF-7 proliferation, whereas MCF-7 proliferation was significantly inhibited by si-LINC01235 (*p* < 0.001; Figure 6B). Meanwhile, the wound-healing assay revealed that pCDH-LINC01235 significantly enhanced MCF-7 migration, and si-LINC01235 showed a notably slower scratch closure rate than control cells (Figures 6C,D), which revealed that silencing LINC01235 inhibited MCF-7 migration (*p* < 0.001). Furthermore, the transwell assay demonstrated that LINC01235 knockdown MCF-7s displayed significantly lower invasion potential than the control cells (*p* < 0.001; Figures 6E,F). Collectively, these results suggest that the expression level of LINC01235 affected the proliferation, migration, and invasion of breast cancer cells.

### LINC01235 Negatively Regulates ER Breast Cancer-Related Gene ESR1

**FIGURE 7:**
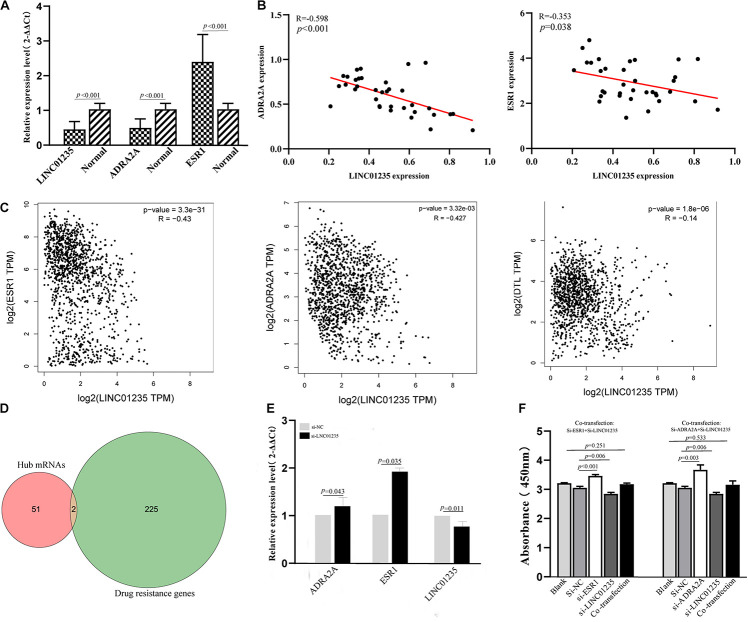
**(A)** Real-Time Quantitative Reverse Transcription Polymerase Chain Reaction analysis was conducted to assess the expression levels of LINC01235, ESR1, and ADRA2A in 35 paired breast cancer tissues and their corresponding adjacent normal tissues. The data were shown as mean ± standard deviations. **(B)** The correlation between the expression of LINC01235 and the expression of ESR1 and ADRA2A. **(C)** The expression correlation between LINC01235-ESR1 and LINC01235-ADRA2A through clinical samples. The correlation between the expression of LINC01235 and the expression of ESR1, DTL, and ADRA2A using GEPIA (http://gepia2.cancer-pku.cn/#index). **(D)** Venn diagram shows the overlapping mRNAs of hub mRNAs in the PPI network and breast cancer drug resistance mRNAs. **(E)** Expression change of ESR1 and ADRA2A after silencing expression of LINC01235 in the MCF-7 cell line. **(F)** The effects of si-ESR1, si-ADRA2A, and co-transfection (si-ESR1, si-LINC01235, si-ADRA2A, and si-LINC01235) on the proliferation activity of MCF-7 cells measured using the CCK-8.

In a previous study, Gonzalez-Malerva found 227 resistant candidate mRNAs when analyzing high-throughput ectopic expression profiles of tamoxifen resistance in breast cancer (Gonzalez-Malerva et al., 2011). As shown in Figure 7D, the Venn diagram indicated that 2/53 (3.77%) of hub mRNAs (ADRA2A and DTL) were overlapped with breast cancer drug resistance genes. In the previous data analysis results, we found that the expression level of LINC01235 was related to the ER status of breast cancer patients. High expression of LINC01235 was significantly correlated with ER positivity (*p* < 0.05, Figure 2C). ESR1 protein is an estrogen receptor 1, and its dysregulation would lead to treatment resistance and metastasis in patients with ER-positive breast cancer. So, is there a correlation between LINC01235 and ESR1? What kind of relationship exists? First, we analyzed the correlation of LINC01235, ESR1, ADRA2A, and DTL by online software (Figure 7C). The results showed that LINC01235 was negatively linked to ESR1 (*R* = −0.43, *p* = 3.3e-31), DTL (*R* = −0.14, *p* = 1.8e-06), and ADRA2A (*R* = −0.427, *p* = 3.32e-03).

The above data indicated a strong correlation among LINC01235, ESR1, and ADRA2A. In order to further verify the correctness of the analysis results, we performed qPCR and correlation analysis on 35 breast cancer clinical samples (Figures 7A,B). Expectedly, the analysis results were consistent with the online prediction results. In the next step, we further analyzed the expression relationships among LINC01235, ESR1, and ADRA2A through cell experiments *in vitro*. The expression of LINC01235 was knocked out in MCF-7 cells, and the qPCR results showed that the expression of ESR1 and ADRA2A was significantly increased (Figure 7E). These further verified the above correlation analysis results. On the other hand, MCF-7 was transfected with si-ESR1 and si-ADRA2A, respectively, and then LINC01235 was silenced. CCK8 results showed that the cell proliferation was increased in the si-ESR1 and si-ADRA2A group and was significantly reduced in the si-LINC01235 group. However, there was no significant change in the cell proliferation rate in the co-transfection group (Figure 7F). These indicated that silencing ESR1 or ADRA2A could noticeably reverse the effect of LINC01235 knockdown on the proliferation.

**FIGURE 8:**
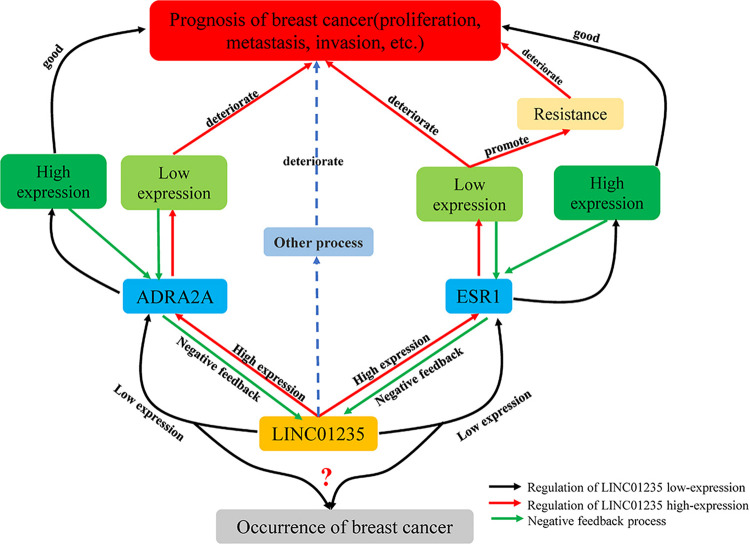
Regulation of LINC01235-ADRA2A and LINC01235-ESR1 in breast cancer.

Based on the above experimental analysis, there may be a reverse regulation mode among ESR1, ADRA2A, and LINC01235. As shown in Figure 8, low expression of LINC01235 regulates the biological stability of ESR1 and maintains the stability of ADRA2A, leading to a good prognosis of patients. The high expression of LINC01235 leads not only to ESR1 imbalance and drug resistance, but also to abnormal expression of ADRA2A, leading to the deterioration of breast cancer. For breast cancer caused by low expression of LINC01235, this needs to be further explored by more experiments and analysis.

## Discussion

At present, some progress has been made in the diagnosis and treatment of breast cancer, but the prognosis of patients with breast cancer is still not optimistic (Ruddy and Ganz, 2019; Waks and Winer, 2019). In recent years, with the development of high-throughput technology, more and more attention has been paid to the function and regulation of lncRNA in diseases. Especially in tumors, lncRNAs not only act as oncogenes, but also act as tumor suppressors (Choudhari et al., 2020; Statello et al., 2021; Xing et al., 2021). However, there are still a lot of lncRNAs to be studied in breast cancer. Comprehensive analysis of lncRNAs in breast cancer can not only fully understand the new mechanism regulating the progression of breast cancer, but also identify new prognostic factors.

With the continuous maturity of high-throughput technologies, a large number of disease databases have been established. Miecznikowski obtained gene expression profiles of five breast cancers through Gene Expression Omnibus (GEO). Through the comparative analysis of the Cox risk ratio model, many genetic molecular markers were found and the survival rate of patients after receiving treatment was estimated (Miecznikowski et al., 2010; Barrett et al., 2013). Goh used high-throughput data to analyze the interaction network of human genes and diseases and found that the protein products of disease genes showed a tendency to correlate in the interaction network (Goh et al., 2004). This new research method makes research no longer limited to a specific experiment or a series of specific experiments, reduces heterogeneity, improves scientific research efficiency, and reveals the potential biological significance of the data and the value of follow-up research. The clinical treatment process provides suitable diagnostic and therapeutic targets.

In this study, we identified differentially expressed lncRNAs from TCGA–BRCA data and found that FGF14-AS2, LINC01235, AC055854.1, and AC124798.1 were significantly associated with the OS of breast cancer patients. Through univariate and multivariate Cox analysis, LINC01235 and AC124798.1 were determined to be the most significant prognostic factors for breast cancer. QPCR detection confirmed that LINC01235 was identical to data analysis results in breast cancer tissue. Previous studies suggest that LINC01235 is a long-stranded non-coding RNA, located on chromosome 9 p23, which has a characteristic expression pattern because of its inconsistent expression in different tumor tissues (Tang et al., 2019; Xu et al., 2019). Studies have shown that LINC01235 was highly expressed in gastric cancer, and silencing LINC01235 could inhibit the EMT characteristics of cells *in vitro* (Zhang C. et al., 2021). In breast cancer, the researchers found that the expression of LINC01235 was downregulated, and it was speculated that the upregulation of LINC01235 might represent a more invasive phenotype, but this study was not carried out in depth (Vishnubalaji et al., 2019).

According to differentially expressed miRNAs from TCGA–BRCA and LINC01235 targeted miRNA, miR-190b was screened out. According to the report, miR-190b was increased in breast cancer and was involved in proliferation, migration, and invasion of breast cancer cells by targeting MYLIP. miR-190b could be used as a biological indicator of ER-positive breast cancer (Cizeron-Clairac et al., 2015; Zhao et al., 2020). The above studies indicate that miR-190b regulates tumor growth and may be involved in breast cancer progression. Meanwhile, two candidate genes (ADRA2A and DTL) obtained by comparison screening have been reported in cancer. In breast cancer, for patients with luminal tumors, ADRA2A was an independent predictor of DMFS and the only factor that preserves its importance (Rivero et al., 2019). DTL depletion resulted in the disruption of the mitotic proteins cyclin B, Cdt2, CDK1, and aurora as well as the upregulation of the cell cycle arrest gene p21 (Ishii et al., 2010; Slenn et al., 2014).

Based on the prognostic roles of potential lncRNAs using TCGA–breast cancer data, LINC01235 was found to be a favorable prognostic biomarker for patients with breast cancer. In this study, we found that the uncharacteristic expression of LINC01235 in breast cancer tissues was significantly lower than that in normal tissues, and the high expression of LINC01235 was associated with poor prognosis of breast cancer patients. Functional studies have shown that overexpression of LINC01235 (pCDH-LINC01235) could promote the proliferation, migration, and invasion of MCF-7 cells *in vitro*. However, LINC01235 played a tumor suppressor role in breast cancer. Besides, our results demonstrate that expression of LINC01235 was significantly positively correlated with pathological N (*p* < 0.05), tumor stage (*p* < 0.05), and ER status (*p* < 0.05). ER-positive clinical stage samples had elevated expression of LINC01235, so what is the regulatory relationship between the expression of LINC01235 and ER coding genes? ER signal transduction has been widely recognized as an important event in the growth and progression of breast cancer. ER is a transcription factor encoded by ESR1 (Lei et al., 2019; Küchler et al., 2020). Through knockout experiment and bioinformatics analysis, we found that there was a negative correlation between LINC01235 and ESR1. Also, we found that there was a negative correlation between LINC01235 and ADRA2A, the hub gene (ESR1) of drug resistance. Furthermore, we co-transfected si-ESR1 or si-ADRA2A with si-LINC01235 in MCF-7, and then CCK8 results showed that ESR1 or ADRA2A silencing could partially reverse the proliferation effect mediated by LINC01235 knockout. These results indicate that there is a significant feedback loop of mutual inhibition between ADRA2A, ESR1, and LINC01235 in breast cancer cells. Together, our results revealed that LINC01235-ESR1 and LINC01235-ADRA2A could serve as important co-expression pairs in the progression of breast cancer. In the future, more experiments need to be performed to further confirm the roles of LINC01235-ESR1 and LINC01235-ADRA2A in breast cancer.

Based on the current analytical data, low expression of LINC01235 will lead to the occurrence of breast cancer, and high expression of LINC01235 could lead to poor prognosis in patients with breast cancer. All these indicate that there is no correlation between the expression pattern of LINC01235 in breast cancer and survival data. It has been reported that this situation may be caused by the great heterogeneity of TCGA–BRCA samples (Vishnubalaji et al., 2019). Aznar pointed out that the DAPLE gene was low expressed in patients with early stage of colorectal tissues and normal patients, acting as a tumor suppressor, but begins to be high expressed in the late stage of cancer, indicating poor prognosis (Aznar et al., 2015). Additionally, previous studies have found that LINC01235 may be related to immune regulation in cancer (George et al., 2017). Therefore, it is speculated that the bidirectional regulation of LINC01235 may be an immune escape mechanism of breast cancer cells. After tumorigenesis, breast cancer cells may hijack LINC01235 and induce the inactivation of its promoter, methylation sequence, or regulator, thereby evading the immune system. Galanos found that p53 was a regulator of p21. In normal tissue samples, p21 acted as a tumor suppressor. However, when p53 was missing or inactivated, p21 will sharply promote tumor growth and spread throughout the body (Galanos et al., 2016). Moreover, according to our research data, we speculate that it may be caused by the negative feedback mechanism of LINC01235-ESR1 and LINC01235-ADRA2A. The high expression of LINC01235 caused the imbalance of ESR1 and led to drug resistance in patients with breast cancer, resulting in a poor prognosis. Similarly, the high expression of LINC01235 reduced the expression of ADRA2A. As a tumor suppressor gene, the low expression of ADRA2A directly made the prognosis of breast cancer worse. The overall effect may be the same as the data we analyzed; LINC01235 overexpression leads to poor prognosis in breast cancer patients. More experiments are needed to verify the regulation mechanism.

## Conclusion

LINC01235 plays a critical role in facilitating the proliferation, migration, and invasion of breast cancer, and our results demonstrated that the effect is achieved by LINC01235-ESR1 and LINC01235-ADRA2A negative feedback pairs. LINC01235 may act as an effective biomarker for the diagnosis and treatment of breast cancer in the future.

## Data Availability Statement

The original contributions presented in the study are included in the article/Supplementary Material, further inquiries can be directed to the corresponding authors.

## Ethics Statement

The studies involving human participants were reviewed and approved by The Ethical Committee of Shuguang Hospital. The patients/participants provided their written informed consent to participate in this study.

## Author Contributions

ZL and ZQ conducted the data analysis and wrote the manuscripts. FC, LM, and SJ carried out data analysis and manuscript revision. JC designed the study. All authors read and approved the final manuscript.

## Conflict of Interest

The authors declare that the research was conducted in the absence of any commercial or financial relationships that could be construed as a potential conflict of interest.

## Publisher’s Note

All claims expressed in this article are solely those of the authors and do not necessarily represent those of their affiliated organizations, or those of the publisher, the editors and the reviewers. Any product that may be evaluated in this article, or claim that may be made by its manufacturer, is not guaranteed or endorsed by the publisher.

## Footnotes

1
https://tcga-data.nci.nih.gov/

2
http://gepia2.cancer-pku.cn/#index

3
http://starbase.sysu.edu.cn/

4
https://lncipedia.org/

5
http://www.csbio.sjtu.edu.cn/bioinf/lncLocator/

6
http://starbase.sysu.edu.cn/

7
http://carolina.imis.athena-innovation.gr/diana_tools/web/

8
http://miRTarBase.cuhk.edu.cn/

9
https://string-db.org/

10
http://sangerbox.com/Tool

## Supplementary Material

Supplementary Figure 1 **(A)** Kaplan–Meier estimates of the survival of GSE21653 testing-set patients (*n* = 252) and GSE42568 testing-set patients (*n* = 104) using the 2-lncRNA (LINC01235 and AC124798.1) signature. **(B)** ROC of LINC01235 based on GSE21653 testing-set and GSE42568 testing-set.

Supplementary Table 1 The targets miRNAs of LINC01235 were analyzed by DIANA tools.

Supplementary Table 2 The targets mRNAs of miR-190b were analyzed by DIANA tools.

Supplementary Table 3 The same mRNAs between targeted mRNAs and differentially expressed mRNAs.

Supplementary Table 4 53 mRNAs in the protein–protein interaction network.
