# Supplementary figures and images for: Identification of Key lncRNA–mRNA Pairs and Functional lncRNAs in Breast Cancer by Integrative Analysis of TCGA Data
Source: Front Genet. 2021 Aug 20;12:709514. doi: 10.3389/fgene.2021.709514 (PMC8417727; doi:10.3389/fgene.2021.709514)

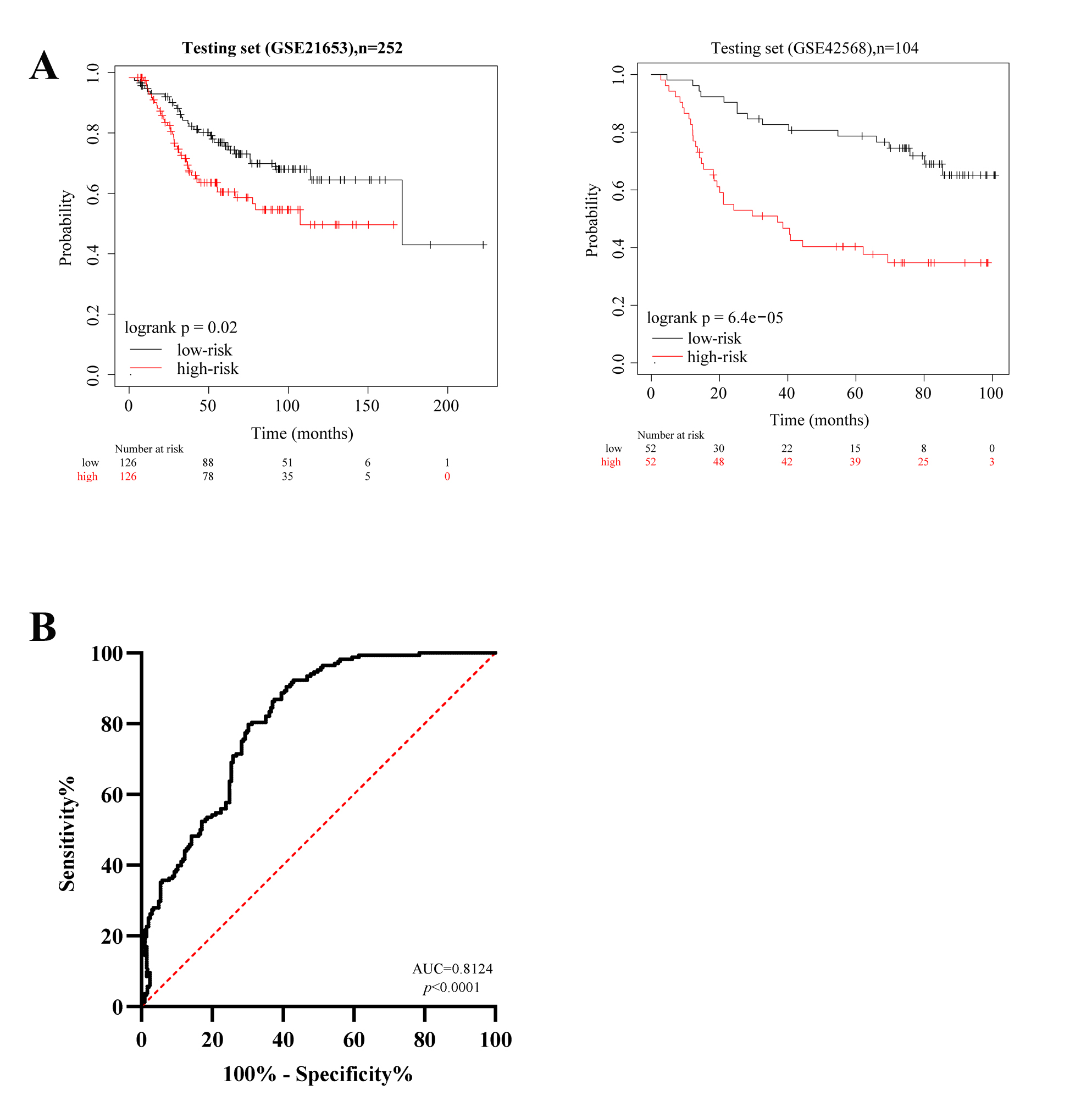

Supplement: Supplementary Figure 1 — (A) Kaplan–Meier estimates of the survival of GSE21653 testing-set patients (n = 252) and GSE42568 testing-set patients (n = 104) using the 2-lncRNA (LINC01235 and AC124798.1) signature. (B) ROC of LINC01235 based on GSE21653 testing-set and GSE42568 testing-set. [file Image_1.JPEG]
