# Supplementary material for: Identification of Key lncRNA–mRNA Pairs and Functional lncRNAs in Breast Cancer by Integrative Analysis of TCGA Data
Source: Front Genet. 2021 Aug 20;12:709514. doi: 10.3389/fgene.2021.709514 (PMC8417727; doi:10.3389/fgene.2021.709514)
Supplement: Supplementary Table 3 — The same mRNAs between targeted mRNAs and differentially expressed mRNAs. [file Table_3.docx]

Supplement 3. The same mRNAs between targeted mRNAs and differentially expressed mRNAs.

| CXCL2,MME,MYH11,DMD,KLF15,FGF2,PI15,PDE3B,PTGS2,SEMA3D,CAV2,EBF1,LIFR,EBF3,SYNE3,PID1,CSRNP3,NR3C2,SEMA6D,GNAI1,FOXP2,MTURN,NFIB,SH3BGRL2,,PPP1R12B,S1PR1,PELI2,RCAN1,CPEB1,NPR3,TMEM47,SEMA3A,IGF2,ATP1B2,EMP1,PRUNE2,PTCH1,LRIG3,ADRA2A,CFL2,ZDBF2,NOV,CAT,ERG,BACH2,TRIM2,KCTD12,HOXA10,SAMD4A,NAV3,EEPD1,BCL11A,PM20D2,HPGD,CPM,NFASC,PTHLH,RGL1,SYNPO,CYBRD1,MPP6,SORBS2,BTBD11,UST,CEBPA,LRCH2,DIXDC1,FOXN3,AK4,PPARA,DSEL,NECTIN4,ERBB3,GATA3,SLC7A11,CDC7,ADAM19,GRHL2,CDH2,ELAVL2,GOLT1A,OVOL1,CLGN,MEX3A,HASPIN,PRR15L,CDC25A,LEF1,FNDC1,PPP2R2C,DIAPH3,GPRC5A,HIST1H2BC,CEMIP,OLR1,MND1,P4HA3,IQANK1,ZNF695,KIAA1211,SPC25,DTL,DEPDC1,NEIL3,MMP1,COL11A1 |
| --- |
