# Supplementary material for: Identification of Key lncRNA–mRNA Pairs and Functional lncRNAs in Breast Cancer by Integrative Analysis of TCGA Data
Source: Front Genet. 2021 Aug 20;12:709514. doi: 10.3389/fgene.2021.709514 (PMC8417727; doi:10.3389/fgene.2021.709514)
Supplement: Supplementary Table 4 — 53 mRNAs in the protein–protein interaction network. [file Table_4.docx]

Supplement 4. 53 mRNAs in the protein-protein interaction network.

| ADAM19，PTHLH，PID1，ADRA2A，CXCL2，S1PR1，GNAI1，BACH2，EBF1，BCL11A，CAT，CFL2，PPARA，PTGS2，CDC25A，CUX1，CDC7，DTL，CDH2，FGF2，LEF1，SEMA3A，NFASC，CEBPA，KLF15，GATA3，MYH11，CPM，MME，PTCH1，FOXP2，MMP1，UST，DEPDC1，MND1，NEIL3，SPC25，DIAPH3，DMD，DSEL，ERBB3，GRHL2，IGF2，HOXA10，GOLT1A，PPP1R12B，OVOL1，HPGD，NR3C2，NOV，NPR3，RGL1，RCAN1， |
| --- |
